# Glass Transition and Crystallization of Chitosan Investigated by Broadband Dielectric Spectroscopy

**DOI:** 10.3390/polym17202758

**Published:** 2025-10-15

**Authors:** Massimiliano Labardi, Margherita Montorsi, Sofia Papa, Laura M. Ferrari, Francesco Greco, Giovanni Scarioni, Simone Capaccioli

**Affiliations:** 1 Consiglio Nazionale delle Ricerche (CNR), Istituto per i Processi Chimico-Fisici (IPCF), Sede Secondaria di Pisa, Largo Pontecorvo 3, 56127 Pisa, Italy; margheritamontorsi@cnr.it (M.M.); simone.capaccioli@unipi.it (S.C.); 2 College of Physics and Optoelectronic Engineering, Shenzhen University, Shenzhen 518060, China; 3 The Biorobotics Institute and Department of Excellence in Robotics & AI, Scuola Superiore Sant’Anna, Viale R. Piaggio 34, 56025 Pontedera, Italy; sofia.papa@santannapisa.it (S.P.); laura.mferrari@santannapisa.it (L.M.F.); francesco.greco@santannapisa.it (F.G.); 4 Interdisciplinary Center on Sustainability and Climate, Scuola Superiore Sant’Anna, Piazza Martiri della Libertà 33, 56127 Pisa, Italy; 5 Physics Department, University of Pisa, Largo Pontecorvo 3, 56127 Pisa, Italy; g.scarioni@studenti.unipi.it

**Keywords:** chitosan, glass transition, water, crystallization, dielectric spectroscopy

## Abstract

Chitosan films obtained by solution casting were investigated by broadband dielectric spectroscopy (BDS) to explore both their glass transition and the effects of thermal annealing on molecular dynamics, deriving from residual water content as well as from cold crystallization. Glass transition at low temperatures could be evidenced in as-produced as well as thermally annealed films, where non-Arrhenian dielectric relaxation processes, consistent with a structural (α) relaxation, could be detected. The process detected at low temperatures could reflect the dynamics of residual water slaved by the polymer matrix. Secondary (β) relaxations, along with a slow process ascribed to interfacial polarization at the amorphous/crystalline interfaces, were concurrently detected. In most cases, a further Arrhenian process at intermediate temperatures (α_c_) was present, also indicative of crystallization. Notably, the α processes, due to the primary relaxation of the polymer matrix plasticized by water, could be discriminated from other processes, present in the same frequency range, thanks to improvements in the dielectric fitting strategy. All relaxation processes showed the expected dependence on *T*_a_. The more accurate exploration of the glass transition for chitosan helps to better rationalize its crystallization behavior, in view of an optimized application of this biopolymer.

## 1. Introduction

There is renewed interest in investigating the properties of chitosan, as a low-cost, bio-derived, and bio-degradable polysaccharide material with potential applications in the fields of energetics and sensors [1]. Recently, it has garnered growing attention due to its newly demonstrated piezoelectric properties [2]. Chitosan can be easily produced from natural chitin, the most abundant biopolymer after cellulose, extracted from marine crustaceans, insect cuticles, and microorganism cell walls.

**Figure 1:**
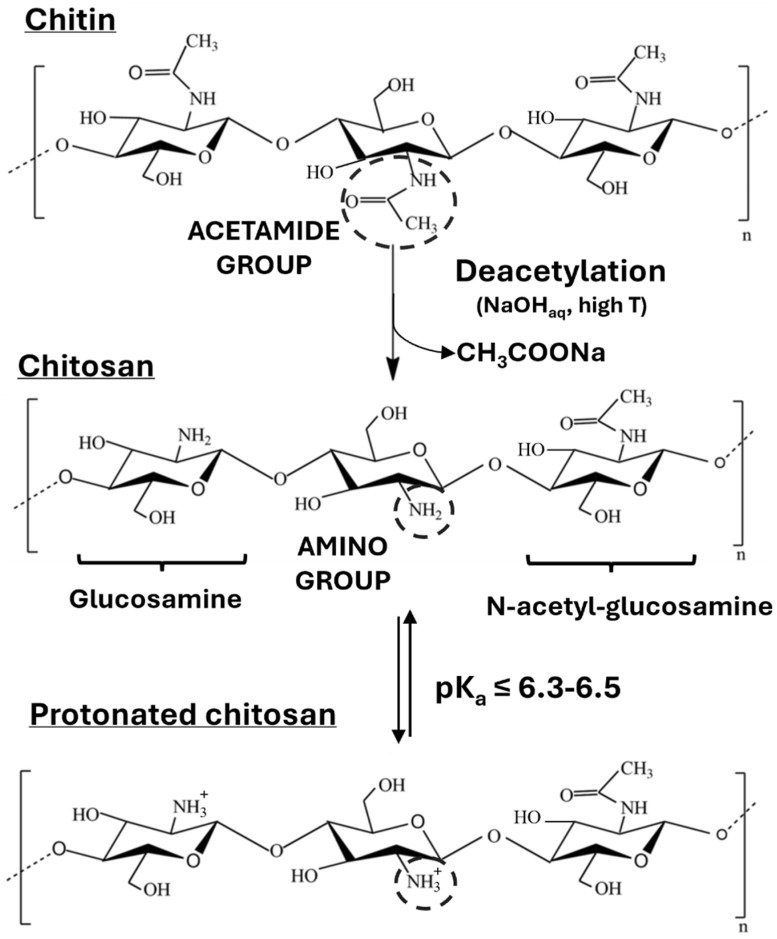
The chemical structure of chitin, chitosan, and protonated chitosan in a slightly acidic environment. In the deacetylation process, the N-acetylglucosamines are converted into glucosamine units, which have a primary amino group (-NH_2_) instead of the secondary acetamides (-NHCOCH_3_). The deacetylation reaction occurs with sodium hydroxide (NaOH) at high temperatures.

Since chitin is not water-soluble, its conversion to chitosan is also a convenient way to improve its processability, due to the increase in solubility in a slightly acidic environment. Indeed, the presence of amine groups (-NH_2_), which replace most acetyl groups (-NHCOCH_3_) of chitin after a deacetylation reaction, makes the protonation of the chitosan chain accessible in acidic aqueous environment (pK_a_ < 6.3–6.5). The transformation of chitin into chitosan through deacetylation and the subsequent protonation process is illustrated in Figure 1.

The protonation of chitosan chains increases the interchain electrostatic repulsion, leading to a faster dissolution of chitosan in water and other polar solvents. After processing, chitosan can be reverted to chitin through the inverse acetylation reaction, or deprotonated and thus compactified by neutralizing the amine groups with an alkaline solution. Due to its highly polar nature, resulting from the -NH_2_ and -OH groups along the saccharide chains, chitosan can absorb and retain a significant amount of water. The amount of residual water within the chitosan structure influences its mechanical, electrical, and piezoelectric properties. Excess water prevents crystallization, while a moderate water content serves as a hydration layer, allowing for the formation of a hydrated crystalline structure, known as the “Tendon” polymorph. In this polymorph, the chitosan polar groups establish strong hydrogen bonds with the interchain water molecules [3]. Further removal of water favors the passage to more compact crystalline forms. The possibility of forming crystals, as well as transitioning into a different crystal phase, can be driven by chemical treatment and thermal annealing [3,4]. Additionally, the type of acid used to dissolve chitosan during solution preparation is key in determining its final crystallinity. Indeed, when acting as counterions, the structure or size of acids can impact intramolecular and intermolecular interactions [5]. The piezoelectricity of hydrated and anhydrous chitosan crystal forms is currently under study [6].

Despite the interest and efforts in investigating chitosan and its properties, evidence of a glass transition for this polymer is not well established. Differential scanning calorimetry (DSC), dynamic mechanical thermal analysis (DMTA), and broadband dielectric spectroscopy (BDS) are often used to identify a glass transition. Most works in the literature that report a glass transition typically describe a relatively high glass transition temperature (*T*_g_) [7,8,9]. In contrast, others do not observe any behavior that can be attributed to a glass transition [10]. Typically, *T*_g_ in the range from −23 to 203 °C has been reported, often much higher than room temperature, where hydration water appears to act as a plasticizer, therefore decreasing *T*_g_ [9]. For instance, Qiao et al. [7] reported an interval of *T*_g_ from 40 to 115 °C, with the variability depending on the water content, as inferred from weak features visible in temperature-modulated (TM)DSC thermograms. These results are more reliable among others, since TMDSC is less sensitive to melting enthalpy, and therefore more able to highlight glass transitions compared to melting/crystallization transitions. In other DSC studies, the water content influence has been investigated, reporting *T*_g_ = 30 °C for water content ranging from 8 to 30% [11]. DMTA studies reported a *T*_g_ from −23 to 72 °C depending upon moisture content [12]. In other works, *T*_g_ derived by DSC ranges from 18 to 62 °C [9], and from 52 to 84 °C [13,14,15]. Another study by DSC observed a variation in *T*_g_ in the range from 18 to 56 °C, depending on the various acids used [16], while values of *T*_g_ from 140 to 150 °C were reported elsewhere by DMTA [8,17] and DSC [8], as well as a higher value of 203 °C by both techniques [18]. Values of *T*_g_ = 18 °C or *T*_g_ = 28 °C were inferred by BDS conductivity measurements [9], while *T*_g_ = 54 °C was derived by high-frequency BDS [19]. Then, overall, there is poor consensus on the value of *T*_g_ in chitosan, whereas evidence of the actual occurrence of vitrification is also weak. Indeed, slight changes in DSC thermograms reported in most of the literature and ascribed to a glass transition could likewise be due to melting or crystallization processes that could also be influenced themselves by water content. In any case, BDS studies of the chitosan glass transition are not numerous. It is noteworthy to mention that only careful analyses based on multi-observable techniques can help to infer information on a complex scenario that often characterizes the dynamics of biomacromolecular systems.

Primary relaxation in semicrystalline polymers has been observed to have multiple sources. The main one, characteristic of amorphous polymers, and of glass-former materials in general, is due to the amorphous fraction of the material. It is the one that is more appropriately related to glass transitions. We will refer to such a process as α-amorphous (α_a_), or simply α process. The second one is ascribed to chain portions partly belonging to a crystal, and partly still in the amorphous state, or to amorphous chains severely constrained among crystals, typically located at the interface between crystal and fully amorphous phases. This region is often referred to as the rigid amorphous fraction (RAF) or the constrained amorphous fraction, and the related relaxation process is commonly known as the α-crystalline (α_c_) process. The same types of dielectric relaxation processes are exhibited for instance by poly (vinylidene fluoride) (PVDF) [20,21], a well-known semicrystalline polymer with piezoelectric and ferroelectric properties, widely used for applications. In the case of this polymer, *T*_g_ is around −40 °C, easily detected by standard DSC [20], where thermograms exhibit a change in specific heat.

In addition to primary relaxations, two main types of different relaxation processes are detectable by BDS. Secondary (or β) relaxations are due to orientation of local dipoles, carried by monomers or side groups. Since their size is small, motion occurs at high frequencies, or in other words, it shows up in the spectral range of BDS at low temperatures. Interfacial polarization (or σ) relaxations are due to mobile charges that accumulate at the interfaces of heterophasic materials or of nanocomposites. Typically, the resulting polarization depends on electrical conductivity and shows up at high temperatures in BDS spectra. These processes are also referred to as Maxwell–Wagner–Sillars (MWS) relaxations, after the scientists that worked out models for their interpretation.

The presence of residual water in chitosan is known to have a plasticizing effect [9], similarly to other polysaccharides and, in general, to other biopolymers. The effect of water freezing below 0 °C has been evidenced by DSC for heavily swollen (hydrogel) chitosan samples [22], as well as, in general, in those cases when the water content is so high to lead to coexistence of hydrated polymer and a considerable amount of free water (e.g., Ref. [7]). However, that is not generally the case. When water content decreases, free water may become confined by the hydrated chains; therefore, its mobility becomes affected by that of the host matrix, and vice versa. That is against the common view that only the dynamics of macromolecules are “slaved” by the dynamics of the solvent, and not the opposite. For instance, it was found that mobility of hydrated t-RNA is still active down to −70 °C [23], which would not be the case if dynamics were ascribed to the macromolecule itself, since size and rigidity of t-RNA molecules would entail much slower dynamics. Instead, what is observed could be the water dynamics slowed down by coupling with macromolecules.

An example of the described scenario was reported in Ref. [24] for a number of oligomers and polymers of biological interest, typically in hydrated form, including polysaccharides like dextran. Dielectric relaxation studies of these materials with different degrees of hydration show the presence of two different Arrhenian processes, along with two different VFT processes. The Arrhenius process at lower temperature is ascribed to a fast relaxation of the solvent (water), while the one at higher temperature is interpreted as a slower relaxation of those solvent molecules interacting with the matrix. A process with non-Arrhenian Vogel-Fulcher-Tammann (VFT) dependence, defined in the Materials and Methods section, ascribed to the structural α-relaxation of the macromolecules, shows up at the highest temperatures. Furthermore, a second VFT process is observed at lower temperatures. The two VFT processes show similar trends in the relaxation plot, appearing as shifted in temperature, so that they could be interpreted as pertaining to two different glass temperatures; actually instead, the VFT process at lower temperature pertains to water “slaved” by the matrix, that is, whose dynamics are slowed down by interaction with the matrix itself, so to reflect in part their own dynamics. Fast water relaxation would be instead that of excess water interacting with the matrix more weakly, for instance, by hydrogen bonding networks. To our knowledge, no such studies were conducted on chitosan so far.

BDS studies of neutralized chitosan obtained from acetic acid solutions, similar to those studied in this work, were reported in 1997 [25]. The temperature was raised to 150 °C to obtain samples with lower humidity. Two processes were observed: the β-relaxation at low temperatures and the MWS relaxation at high temperatures. More detailed studies were performed in 2004 [26], where the same processes were compared for samples with and without neutralization, showing that the β-relaxation remained unchanged, while the MWS relaxation was faster for non-neutralized samples. Furthermore, comprehensive studies of a wide range of different polysaccharides, compared with the corresponding oligosaccharides, but not including chitosan, have identified up to five different secondary relaxation processes [27,28], which, however, still do not include a clear primary, or structural, relaxation (α process). Such relaxations were classified as follows, in order of increasing temperature:

(i) γ-relaxation, due to the motion of the side groups of the glucosamine units;
(ii) β-relaxation, due to the motion of the glucosamine units linked by the glycosidic bonds;
(iii) β_wet_-relaxation, a modified β-relaxation due to hydrogen-bonded water, strongly depending on water content;
(iv) δ-relaxation, at intermediate temperature;
(v) σ-relaxation, influenced by ionic conductivity.

All these processes presented an Arrhenius temperature dependence. Later, relaxations (iii) and (iv) were related to the same process [27].

The γ-relaxation appears usually at higher frequencies than available with our setup, so it could not always be detected here. The δ-relaxation is named here as α_c_ (α-crystalline), for reasons that will be explained later. Finally, the σ-relaxation is also referred to here as “slow.”

The limited and weak evidence of structural α-relaxation in chitosan by dielectric spectroscopy so far, and the lack of information about the role of crystallization in the molecular dynamics of thermally annealed chitosan films, have led us to look for the evidence of a glass transition in the low-temperature region, along with the one at higher temperature, not reported to date. Comparison of our evidence with the existing literature data has allowed us to improve the interpretation of the observed dielectric relaxation processes. In particular, we have identified two different β-relaxations (referred to as β-fast and β-slow) and two structural relaxations (α-fast and α-slow), where the fast processes are found at lower temperatures and could be consistent with those of hydration water slaved by the polymer chains, as elucidated in Ref. [24], while the slow ones, at higher temperatures, represent more properly the dynamics of the polymer matrix, which can be plasticized by water, hence these processes will be also referred to as β and α_a_, respectively. At even higher temperatures, dielectric relaxation is primarily influenced by conductivity effects that often show up as overlapped to the structural relaxation of this polymer. Therefore, the structural process, that is, the one related to its glass transition, can be hardly discriminated from conductivity-related processes by resorting to BDS.

## 2. Materials and Methods

Preparation of chitosan films. Chitosan (Sigma-Aldrich, St. Louis, MO, USA) of medium molecular weight was mixed 1% (*w*/*v*) with acetic acid at 2% (*v*/*v*) dilution in a water solution. For comparison, samples were prepared from solutions of formic and lactic acids with the same concentration, 2% (*v*/*v*). The solutions were stirred overnight and then centrifuged at 3500 rpm for 1 h. The supernatant solution was collected, to remove impurities, and poured into circular Petri dishes (45 mm diameter) using 6 mL of solution for each cm^2^ of deposition surface, calculated to achieve a final dry film thickness of approximately 30 µm. After 6 h in the oven at 60 °C, the films were left to dry overnight at room temperature. Once dried, they were immersed in a 1 M NaOH solution bath for neutralization for different amounts of time, namely, 30, 60, and 90 min. Non-neutralized films were used as controls and hereafter indicated as A0, when acetic acid was the chitosan solvent (F0 for formic acid, and L0 for lactic acid). Neutralized films are henceforth indicated as A30, A60, and A90 (F30, F60, F90, L30, L60, and L90 as comparison samples). After this step, the films were rinsed with distilled water, and their pH was measured using pH indicator strips until it reached a value of 7. Once the films were washed, they were placed on a flat surface (i.e., the bottom of a Petri dish) to let them dry at room temperature for 3 h, resulting in flat, thin films with a thickness of around 30 μm.

To study the effect of thermal annealing, all films (unless specified) were initially measured as produced. However, thermal annealing was also implicitly carried out during dielectric measurements, where temperature ramps are used to investigate the temperature dependence of molecular dynamics. Therefore, only the measurement during the first heating ramp can be considered to concern the hydrated sample, while the following ones are made on samples that should be considered as previously annealed at least up to 100 °C. Comparison measurements were also performed on pre-annealed samples, at temperatures of 100 °C or 140 °C. The residual water content of our samples before measurements was estimated by TGA around 12% for non-neutralized, and 10% for neutralized samples (Figure S1 of the Supplementary Materials), in accord with most of the literature.

**Figure 2:**
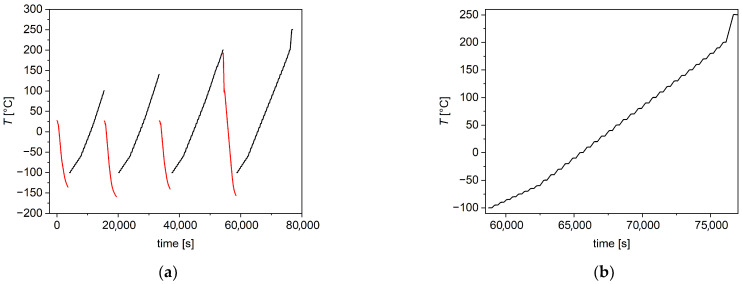
Temperature ramps used for BDS measurements. (**a**) Complete temperature cycle. Red curves are cooling ramps, while black curves are stepped heating ramps, when isothermal measurements are performed during the constant temperature periods. (**b**) Detail of a heating ramp, where isothermal steps are visible.

Dielectric spectroscopy. Broadband dielectric spectroscopy (BDS) was performed by an Alpha Analyzer spectrometer (Novocontrol technologies GmbH & Co, Montabaur, Germany), equipped with a Novocontrol Quatro nitrogen gas flow cryostat. The produced chitosan films, with an approximate thickness of 30 μm, which were not previously subjected to thermal annealing, were sandwiched between two gold-plated discs (diameter 12 mm; thickness 0.88 mm) and inserted into the BDS measurement cell. A metal spring washer was included to ensure mechanical stability during temperature ramps. Isothermal measurements were performed by heating at 5 °C or 10 °C temperature steps, ranging from 0.1 Hz to 1 MHz. Each isotherm lasted for about 10 min. To obtain results for different annealing temperatures, the following thermal cycling was used: cooling ramps were made down to at least −100 °C, while heating ramps were made up to increasingly higher final temperatures (100 °C, 140 °C, and 200 °C), in such a way that the following measurement concerned the same sample annealed at increasingly high temperatures. A sketch of performed thermal cycles is presented in Figure 2. The same kind of thermal treatment was applied in Ref. [29]. For one of the neutralized samples, labeled A90(2), pre-annealing at 100 °C for 1 h was performed, and afterwards, the heating ramps (after cooling performed as for the previous sample) were made twice up to 250 °C. This was performed to study the effect of annealing at 250 °C. All BDS measurements were performed in a nitrogen atmosphere.

Data analysis. Relaxation plots were obtained by fitting dielectric spectra by the Grafity freeware (grafitylabs.com, ver. 0.5.5), by using the Havriliak–Negami (HN) dielectric function for the β, α, “slow” and other occurring processes (indexed by *j*):

(1) $${\Delta \varepsilon }_{\mathrm{HN},j}=\frac{{∆\varepsilon }_{j}}{{\left(1+{\left(i\frac{f}{f_{0,j}}\right)}^{a_{j}}\right)}^{b_{j}}}$$

where Δ*ε* is the dielectric strength, *f*_0_ the relaxation frequency, *a* the width exponent, and *b* the symmetry exponent of the process.

The conductivity contribution *ε*_c_ to the dielectric function can be described by the term

(2) $$\varepsilon_{c}(f)=\frac{\sigma_{0}}{\varepsilon_{0}{\left(i2\pi f\right)}^{n}}$$

that expresses the frequency dependence of conductivity in disordered systems, where *σ*_0_ is the *dc* conductivity and *n* is the conductivity exponent, which identifies different conduction mechanisms [30].

Typically, the dielectric function imaginary part data (*ε*″) are used for fitting, which facilitates identification of relaxation processes that appear as peaks in *ε*″, and allows for graphically visualizing the contribution of single processes, which are barely summed up since εHN″=∑jImΔεHN,j+Imεc. However, the conductivity contribution to *ε*″ becomes often overwhelming, especially in the presence of solvents facilitating transport of ionic species, and at the highest temperatures where conductivity is enhanced.

A representation useful to better evidence relaxation peaks is in terms of tan*δ* = *ε*″/*ε*′. This representation has the further advantage to be independent from thickness variations in the sample, which can happen due to vitrification or crystallization. The global fitting function has the form

(3) tanδ=∑jImΔεHN,j+Imεcε∞+∑jReΔεHN,j+Reεc

that is not a bare sum of terms like in the case of *ε*″. This function contains information on both real and imaginary parts of the dielectric function; therefore, it could be expected that its accuracy for fitting of experimental data could be improved compared to that of the *ε*” alone. The disadvantage of this fitting function is that, while performing the fittings, its different terms cannot be visualized separately, since it is not in the form of a simple summation of terms. Then, it is convenient to perform fittings of *ε*″ first, to estimate general trends of single processes more easily, and then perform a second, refined fitting by using tan*δ*. For our chitosan samples, conductivity contributions were usually rather high, and thickness variations in the sample during BDS measurements were also remarkable. Furthermore, the compresence of different processes (up to four) in the higher temperature regime demanded the highest confidence in the fitting procedure adopted; therefore, it was necessary to resort to the analysis of tan*δ* spectra in order to obtain more reliable results.

The best fits were usually made as follows. First, the value of the unrelaxed dielectric constant *ε*_∞_ was evaluated from the measured *ε*′ value at the lowest temperature, typically –100 °C, and the highest frequency, 1 MHz. Then, the α-fast process, the only one present at the lowest temperatures for most samples, was fitted by imposing *b* = 1 and including the conductivity contribution, if present. For the A0 sample with no annealing, the β-fast process was also included, imposing *b* = 1. For the β-fast process, relaxation frequencies as a function of *T* are best fitted to an Arrhenius function, that is, log *f* = log *f*_∞_ − *E*_a_/(2.3 *k*_B_ *T*) (with *k*_B_ the Boltzmann constant, and *T* in degrees K), providing the values of activation energy, *E*_a_, and relaxation frequency at infinite temperature, *f*_∞_. This is typically performed from *T* = −100 °C to temperatures where the β-fast relaxation peak maximum remains within the measurement spectral window. To improve fittings at higher temperatures, where slower processes appear in the spectra, the β-fast relaxation is still included in the fitting function, by imposing its relaxation frequency as the one extrapolated from the Arrhenius fitting of such process at lower temperatures.

A similar approach is followed for the α-fast process, but in this case, the relaxation frequencies as a function of *T* are fitted to a Vogel–Fulcher–Tammann (VFT) function:

(4) $$f_{\mathrm{VFT}}=f_{\infty }e^{-\frac{B}{T-T_{0}}}$$

where *T*_0_ is the Vogel temperature, i.e., the temperature for which the relaxation time *τ* = 1/2π*f* tends to infinity. This fitting can be made by letting *f*_∞_ as a free parameter, or by fixing *f*_∞_ to coincide with that of the secondary relaxation, when available, in such cases when the structural relaxation tends to merge with the β-relaxation at increasing temperatures, which happens when the two processes have some degree of coupling [31], and then deriving the values of *B* and *T*_0_. The glass transition temperature *T*_g_ is assumed to be the temperature at which Equation (4) takes the value *f*_α_ = 1/(2π*τ*_α_) = 1/(2π 100) Hz, that is, the relaxation frequency assumed by convention to define the glass transition by dielectric measurements [31]. Therefore, *T*_g_ is related to the VFT parameters such as

(5) $$T_{g}=T_{0}+\frac{B}{2.3\;(2+log(2\pi f_{\infty }))}$$

The dynamical fragility index *m* indicates the rapidity of occurrence of vitrification/amorphization with temperature. Fragility is defined as

(6) $$m={\left.\frac{d\;log\tau_{0}}{d\frac{T_{g}}{T}}\right|}_{T_{g}}$$

where *τ*_0_ = 1/(2π*f*_0_) is the relaxation time. By simple calculations, the fragility index can be expressed in terms of the VFT parameters as (all temperatures in degrees K):

(7) $$m=\frac{BT_{g}}{2.3{\left(T_{g}-T_{0}\right)}^{2}}=(2+log(2\pi f_{\infty }))\;(1+\frac{2.3T_{0}}{B}(2+log(2\pi f_{\infty })))$$

At higher temperatures, the α_c_ and α-slow processes, as well as Maxwell–Wagner–Sillars polarization and electrode polarization, are included. All of these processes are fitted by imposing *b* = 1. The α-slow process relaxation frequencies as a function of *T* are then fitted with a VFT function, while the other three processes are fitted with an Arrhenius function.

## 3. Results

**Figure 3:**
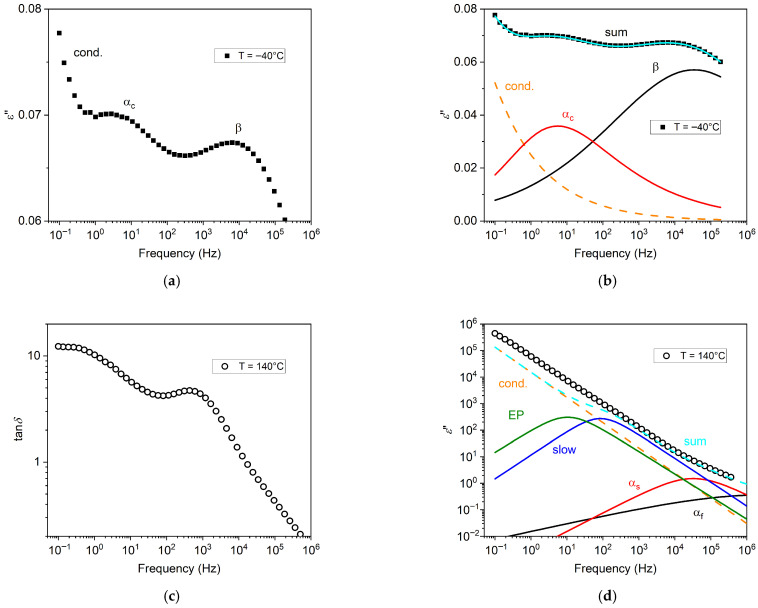
(**a**) Example of broadband dielectric isothermal spectrum at −40 °C for the A90(2) chitosan film annealed at 100 °C. In this illustrative spectrum, the imaginary permittivity *ε*’’ shows a peak corresponding to the secondary process (β) at higher frequency, a peak for the structural process (α_c_) at lower frequency, and a rise due to conductivity (cond.) for the lowest frequencies. (**b**) Fitting functions for the spectrum in (**a**) orange: conductivity (Equation (2)); red: primary relaxation (Equation (1)); black: secondary relaxation (Equation (1)); and cyan: sum of the three contributions. (**c**) Example of tan*δ* dielectric spectrum for the A0 sample annealed at *T*_a_ = 100 °C, for the temperature of *T* = 140 °C. (**d**) The *ε*″ spectrum for the same case. Contributions of single processes as obtained by fitting with the tan*δ* function (Equation (3)) are also reported.

In Figure 3a, an illustrative BDS isothermal spectrum is shown, for the A90 sample annealed at *T*_a_ = 100 °C. Here, two peaks in the imaginary permittivity *ε*″, related to distinct processes, can be easily identified, that is, secondary (β) and primary (α_c_). Conductivity is responsible for the rise at low frequencies. Contributions of single processes are reported in Figure 3b, where their sum provides the total spectrum.

In Figure 3c, the spectral data of tan*δ* = *ε*″/*ε*′ for the A0 sample annealed at *T*_a_ = 100 °C, for the temperature of 140 °C, are shown. In this illustrative spectrum, tan*δ* shows one peak, and a plateau at low frequency. Indeed, by choice of the conductivity function of Equation (2), the low-frequency limit of tan*δ* is a constant value, related to the conductivity exponent *n* [30], and this is confirmed by our measurements at low frequency and high temperatures.

The corresponding data for *ε*″ are instead reported in Figure 3d, showing an almost featureless trend, dominated by conductivity. The contributions of single processes, reported as the colored lines, were obtained by fitting of the tan*δ* spectrum of Figure 3c. The same task could not be easily performed by fitting of the *ε*” spectrum, since it lacks features and therefore allows for more numerous acceptable combinations of contributions obtained by fitting. It is also evident how the fitting result obtained by the tan*δ* function does not correspond exactly to the *ε*″ data. This can be explained by the fact that the thickness of the sample has decreased during temperature ramping, and therefore the *ε*″ values become overestimated, while tan*δ* data are not affected by such thinning. For instance, for the A90(1) sample, the initial thickness was 51 μm, while the final thickness was 32 μm.

By fitting of isothermal spectra at the different temperatures, the logarithm of the HN parameter *f*_0_ (the relaxation frequency) of each process is plotted versus 1000/T (in degrees K), to obtain a relaxation plot. A straight line represents a thermally activated, or Arrhenius, process, and its slope is the activation energy, *E*_a_. A process with a VFT trend appears instead as a convex line, with vertical asymptote at temperature *T*_0_, whereas the dielectric glass transition temperature can be determined as the one where log *f*_0_ = −2.8.

Three types of dielectric relaxation processes are to be expected in a semicrystalline polymer like chitosan:

(1) Secondary (or β) relaxation, ascribed to the motion of glucopyranose rings comprising the polymer chain [27]. The interaction with the environment can influence this process, in particular with other rings from the same polymer chain (intramolecular), from different chains (intermolecular), or with the residual solvent and/or water inside the polymer matrix.
(2) Primary (or α) relaxation, due to the motion of polymer chains, which may be correlated with each other. As temperature decreases, such a correlation may involve larger and larger material domains, leading to structural arrest related to the glass transition. The presence of solvents can also influence this process.
(3) Interfacial polarization relaxation (often named “σ” relaxation), also known as Maxwell–Wagner–Sillars (MWS) polarization, occurring at higher temperatures. It is typically due to the accumulation of free charge carriers at the interface between the amorphous and crystalline regions of materials, at the origin of an additional polarizability, that is, at the base of the properties of nano-dielectrics [32]. This process is also influenced by the mobility of charge carriers, that is, by electrical conductivity.

In addition, electrical conductivity itself and its manifestation in the phenomenon of electrode polarization [31] are expected to affect BDS results, and should be modeled accordingly for reliable interpretation of spectra, as exemplified in Figure 3. In particular, electrode polarization consists of the charge accumulation at the surface of BDS electrodes, similarly to the case of MWS polarization, but with a characteristic size that coincides with the full sample thickness, therefore giving rise to much slower polarization build-up.

**Figure 4:**
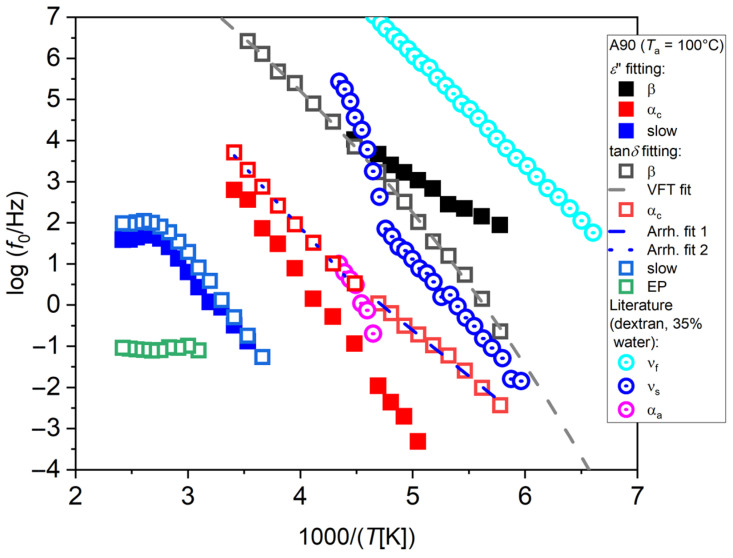
Relaxation plots for the A90 sample with *T*_a_ = 100 °C obtained by fitting of *ε*″ or tan*δ* data. The literature data for dextran, extracted from Ref. [24], are also reported for comparison.

As an example, the relaxation plot for the sample A90 with *T*_a_ = 100 °C is shown in Figure 4. Relaxation frequencies obtained by fitting of *ε*″ and tan*δ* are compared here. Fitting results appear as sensibly different in the two cases. Fitting *ε*″ (full symbols) provides an Arrhenius process at low temperatures, labeled as β, an Arrhenius process at intermediate temperatures, labeled as α_c_, and an Arrhenius process at higher temperatures, labeled as slow, that flattens out at temperatures higher than 100 °C, which coincides with the annealing temperature *T*_a_. By fitting of tan*δ* instead (open symbols), the process at low temperature assumes a slight VFT trend, while the other two processes retain the same behavior, although becoming faster, and a fourth process at high temperature appears, that we assign to the phenomenon of electrode polarization, labeled as EP.

The most remarkable differences are found concerning the relaxations at low temperatures. The β process found by fitting *ε*″ appears instead as a VFT process by fitting of tan*δ*, corresponding to a very low glass temperature (*T*_g_ = −114.7 °C). As stated above, we tend to consider this last result, obtained by fitting of tan*δ*, as more reliable, and therefore our interpretation will be only based on those. It could be hypothesized that this process could represent the dynamics of water interacting with the polymer matrix, and not that of the polymer itself.

To discuss this point, the literature data from Ref. [24] for dextran are shown in Figure 4 (center-dot open circles). In this work, the behavior of the hydrated polymers studied (e.g., polyvinylpyrrolidone) is found to be similar to that of hydrated proteins. In general, along with the polymer relaxation—typically exhibiting a secondary and a primary relaxation, named slow solvent (ν_s_) and α_a_, respectively, where for the latter water acts as a plasticizer lowering the polymer *T*_g_—the relaxation of water is also evidenced, which appears as slowed down by the interaction with the polymer matrix. Such relaxation has a crossover above *T*_g_, where the same process starts to mimic the primary relaxation of the polymer, since it is due to water molecules “slaved” by the polymer matrix [24]. Furthermore, a secondary relaxation of water that is slowed down compared to that of free water is also present, named fast solvent (ν_f_), due to local motions of those water molecules not belonging to the polymer hydration layer, but still constrained by either hydrogen bonding or confined within the polymer chains. Although the type of polysaccharide is different and its hydration level (35%) is higher than in our case, a qualitative comparison with the case of chitosan can still be attempted. The ν_f_ process of dextran could be related to the chitosan VFT process (β) obtained by fitting of tan*δ*. The Arrhenius part of the slow water process of dextran (ν_s_) could be associated with the slow water process of chitosan (α_c_) obtained by tan*δ*. By close observation of our α_c_ process obtained by the tan*δ* fit, a slope change can be noticed starting from 1000/*T* = 4.5, highlighted by the two straight fitting lines. The high-temperature side could be consistent with a VFT process with low fragility, as with the ones usually observed in chitosan, and therefore could be related to the VFT part of the ν_s_ process of dextran. However, since the polymer *T*_g_ could not be observed in this case since its probably embedded into the high-temperature slow process, it is difficult to determine the expected crossover temperature of our ν_s_ process.

**Figure 5:**
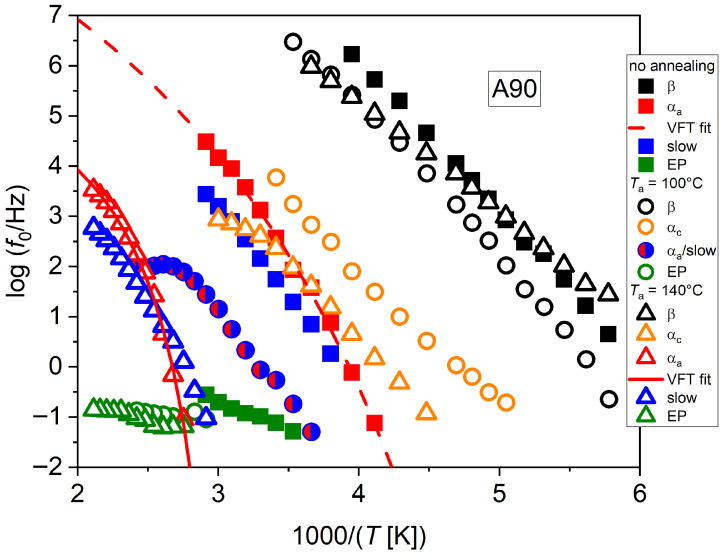
Relaxation plots for the neutralized samples, subjected to multiple annealings up to 140 °C. VFT fittings for the α_a_ processes are also shown.

These comparisons could be improved by the following analysis of the effect of thermal annealing. Figure 5 shows the relaxation plot from the isothermal dielectric spectra of chitosan in the case of neutralized samples, for different values of *T*_a_. The observed behaviors can be summarized as follows. The sample in the initial state, and therefore not annealed, contains a water fraction of around 10%. In this sample, four processes could be identified, as reported in Figure 5 (full symbols), named as β, α-amorphous (α_a_), slow, and electrode polarization (EP). The β process has actually a nearly Arrhenius trend, while the α_a_ process has a VFT trend. The slow process is located very close to the α_a_ one, and the discrimination of these processes was only possible by the adopted fitting of the tan*δ* function, while the attempts made by fitting of the *ε*″ function were usually unsuccessful, since they provided a single process with intermediate characteristics. Actually, in this case the slow process could also be classified as an α_c_ one, being close to the location of the α_c_ process in annealed samples, as shown later. In both cases, though, the existence of this process suggests the possible presence of a crystalline structure even in the pristine material. Finally, a slow process could be detected, only by resorting to tan*δ* fitting, ascribed to electrode polarization, a very well-known background effect in BDS, especially in the presence of high conductivity [31].

For *T*_a_ = 100 °C (same data as in Figure 4), the water content should have decreased, although a residual of a few percent is still to be expected. The detected processes are shown in Figure 5 as the round symbols. The β process becomes slower than in the case of no annealing and assumes a more marked VFT trend. It is therefore likely that the present process can be ascribed to the dynamics of confined water “slaved” by the dynamics of macromolecules [23,24]. An intermediate process appears that cannot be ascribed to the α_a_ one, since by a decrease in water content, the glass temperature is expected to increase. We tentatively label this process as an α_c_ one, due to the supposedly increased crystalline fraction due to thermal annealing. Its trend is an Arrhenius one, although a slight steepening can be noticed at higher temperatures, that could be associated with the phenomenon of slow solvent relaxation observed in Ref. [24], named as ν_s_ in Figure 4.

The α_a_ process could not clearly be resolved in this sample, since it is apparently almost coincident with the slow process. For this reason, it was indicated by the half red/half blue circle in Figure 5. A general feature of slow processes measured by BDS using our thermal cycle (Figure 2) is the slowing down during heating, visible as the plateau starting at a temperature comparable to the one of the last performed annealing. In general, we have observed that this process slows down markedly for increasing *T*_a_. Furthermore, its trend is Arrhenian in specific temperature intervals, while during heating to carry out BDS, transitions are observed where the process tends to slow down or remains steady while the temperature increases. This observation could be consistent with the hypothesis that, by increasing the temperature up to those values where the sample was previously annealed, the crystalline structure remains fairly stable, while when the temperature is increased above such values, further crystallization could be enabled; concurrently, electrical conductivity could decrease due to the lower amorphous content, by virtue of the additional crystallization due to heating, in addition to the decreased water content. Such a conductivity decrease could also be evinced directly from fitting the conductivity contributions to the BDS spectra, examined further on. Nevertheless, our indirect evidence of cold crystallization happening during heating ramps seems rather reasonable, although a direct confirmation of the crystal formation should be obtained, e.g., by XRD measurements, which however should be conducted dynamically during temperature ramps for better comparison. The possible manifestation of a glass transition in the temperature ranges reported in the literature seems difficult to be detected by BDS due to the dominance of conductivity effects at such high temperatures; nevertheless, by employing the analysis of tan*δ* spectra, such discrimination became often possible.

At even higher values of *T*_a_, the transition abovementioned happens at higher temperatures, consistently with our hypothesis. We remark, however, that the chitosan material employed in emerging applications is generally expected to operate in humid or even physiological conditions, like in the case of biomedical applications; therefore, properties of hydrated chitosan are especially relevant in practice. Thermal annealing can be used to optimize the crystalline structure, depending on the specific application; however, some degree of re-hydration is also to be expected in the final device.

Also for *T*_a_ = 100 °C, EP was evidenced at even higher temperatures because of the abovementioned decrease in conductivity due to thermal annealing.

For *T*_a_ = 140 °C, with the water content even more reduced, the β process assumes again an Arrhenius trend, with a relaxation frequency consistent with the previous case at the highest temperatures, while becoming faster at the lowest ones, overlapping to the case of no annealing. This behavior with *T*_a_ seems a general one, also from our observations in chitosan samples produced by formic and lactic acid solutions.

An intermediate process (α_c_) is present that appears as slowed down compared to the one in the case of *T*_a_ = 100 °C. The α_a_ process, although still overlapped to the slow process, could be distinguished in this case by the fitting of tan*δ*. The VFT fitting for the latter is also shown in Figure 5. Finally, an EP contribution was also present. Both slow and EP processes show a slowing down with increasing *T*_a_, consistently with the decrease in conductivity related to both crystallization and water removal. We emphasize that the processes evidenced by our measurements could be detected even simultaneously in some samples. In particular, the α_a_, slow, and EP processes together with the conductivity could be derived from fitting the same spectrum. This contrasts with some of the literature, where only one of the two processes was identified, either the δ (that here we name α_c_) or the σ (slow) process. However, this could be due to the different fitting procedures being adopted.

The values of the activation energy of the Arrhenian processes, and of the VFT parameters and related *T*_g_ and fragility index, are reported in Table 1 for all samples and annealing temperatures. The trend of *T*_g_ with the annealing temperature confirms plasticization of the polymer dynamics by residual water. For the neutralized sample, *T*_g_ shifts from the initial value of −43.0 °C with no annealing (water content of around 10%) to 79.2 °C with *T*_a_ = 140 °C, where the water fraction should be of only a few %. For the non-neutralized sample, analyzed further on, *T*_g_ shifts instead from −31.8 °C (water content 12%) to 54.9 °C (water content higher than in the neutralized case). It is evident that plasticization is not the only effect to rule the polymer dynamics, where the role of hydration water may depend on the crystalline structure as well as the hydrogen bonding network present in each situation. Furthermore, it is noteworthy that the dynamic fragility index found for structural relaxations in the samples with no annealing (9.7 for sample A0, and 34.5 for sample A90) is lower than the ones usually reported for amorphous mono- or polysaccharides [33], that indicates systems with reduced cooperative motions and/or confined environment, but increases with the annealing temperature up to 130.9 (sample A0, *T*_a_ = 140°C) and 235.7 (sample A90, *T*_a_ = 200°C), indicative of a more fragile glassy behavior.

Relaxation plots for the A90 sample annealed up to 250 °C are shown in Figure S2 of the Supplementary Materials. The resulting effects at such high temperatures could be influenced by the tendency of the formed crystals to melt, or at least become more disordered, and of the polymer itself to degrade. Remarkably, indeed, as *T*_a_ is increased, we note that the appearance of the film changes drastically, becoming very dark and brittle after annealing at 250 °C, probably due to progressive thermal degradation. Indeed, the onset of chitosan decomposition already starts at 210 °C, as documented by thermogravimetric analysis combined with mass spectrometry [10].

Considering that the value of *E*_a_ of secondary processes could provide information on the degree of bonding and consequent immobilization of polymer segments or side groups, a correlation with annealing temperature could elucidate the role of hydrogen bonding or interchain interactions in the formation of complex crystalline structures in these materials. Therefore, BDS results could be a useful complement to structural data from XRD as well as to calorimetric analyses.

**Figure 6:**
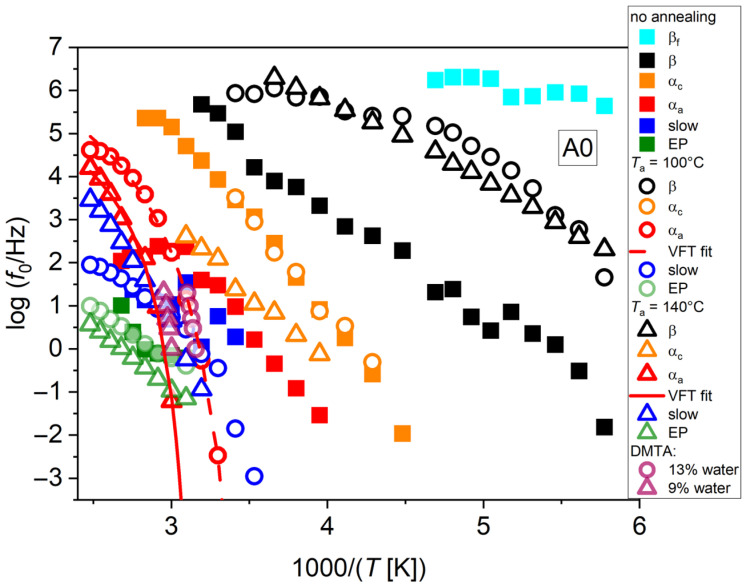
Relaxation plots for the A0 sample, with no neutralization treatment, subjected to multiple annealings up to 140 °C, with added DMTA data from the literature (data extracted from [12], Copyright 2002, with permission from Elsevier). VFT fittings for the α_a_ processes are also shown.

The same study was conducted on non-neutralized films, that is, not subjected to alkaline treatment, to provide evidence of the effect of neutralization. In Figure 6, the related relaxation plots are shown. The same kinds of relaxation processes as in the neutralized case are present, apart from the appearance of a fast process (β_f_ in Figure 6) in the case of no annealing, that could not be detected in the neutralized case, which could be classified as a ν_f_ process according to Figure 4. The corresponding β process is also strongly slowed down and with a nearly Arrhenius trend.

The slow process in non-neutralized samples exhibits a different trend with respect to *T*_a_ compared to the neutralized one. Namely, such a process is faster compared to that of neutralized samples, consistently with the higher conductivity of these samples, and an increase in *T*_a_ slows down the process for *T*_a_ = 100 °C, where also the tendency to slow down due to crystallization already seen for the A90 sample is present. However, this process speeds up again by further increasing *T*_a_ to 140 °C. In this case, a smaller rate of crystal growth than in the neutralized samples could be hypothesized, probably because crystal growth is expected to be more difficult in the presence of protonated amines, giving rise to this different behavior.

A comparison with the literature data provides hints for the interpretation of relaxation processes detected in our samples. In particular, it is remarkable that DMTA data from Ref. [12] for non-neutralized materials with comparable water content to that of our specimens are well overlapped to our α_a_ process, as reported in Figure 6. Upon decreasing the water content, slowing down is also visible, although no exact comparison with our data is possible, since the samples measured by DMTA in Ref. [12] had a minimum water fraction of around 9%.

## 4. Discussion

As seen in the Introduction, values of *T*_g_ of chitosan reported in the literature are usually higher than room temperature, which would entail that crystallization would be hindered because of some degree of structural arrest below *T*_g_. On the contrary, there is evidence by XRD analysis that chitosan crystallization can occur easily even at room temperature, lower than such high values of *T*_g_. Indeed, XRD spectra generally show some degree of crystallinity, depending on the preparation method of the samples [34,35,36,37], whereby post-processing methods can be required to obtain the amorphous material [37]. This has motivated us to explore the occurrence of a glass transition at low temperatures. BDS appeared as a convenient method to explore both the glass transition and the crystallization processes, due to the polar character of chitosan, exhibiting various polar moieties linked to its polysaccharidic chain, namely, amines (-NH_2_), and residual amides (-NH-COOH), along with primary hydroxyl groups (-OH) themselves. Crystallization can be detected as a decrease in the dielectric signal due to the relaxation of the amorphous material, along with the appearance of an additional primary relaxation (α_c_) ascribed to the rigid or constrained amorphous fraction, at the boundary with crystalline regions. Additionally, the detection of the interfacial polarization (or MWS) relaxation may also indicate the presence of a crystalline fraction and the corresponding amorphous/crystalline interface.

Films were obtained by solution casting, where chitosan is dissolved in an acidic solution, resulting in the formation of protonated amines due to the acidic environment. Due to electrostatic repulsion, the charged groups in the polymer prevent its compactification and crystallization when the solvent concentration decreases. Consequently, a post-treatment of the produced films with an alkaline solution for an extended time period is necessary to obtain a more compact material. This process neutralizes the amine groups and helps eliminate residual acid molecules by forming salts, removed through successive washing. Therefore, as-prepared films retain a considerable amount of solvent, since water and residual dissociated acid molecules may remain in the polymer matrix. An excess of solvent may contrast interchain interactions, so that, although the material is mainly amorphous, the structural relaxation due to the correlation of different parts of polymer chains is not evident in the pristine neutralized material, and only the secondary relaxation due to the motion of monomers or side chains is observable.

Information on the crystalline structure can be given by XRD analysis, which for polymers can provide the crystalline phase type, and the degree of crystalline order. However, this aspect is not the main focus of the present paper. Nonetheless, in this work indirect evidence of crystallization can be provided by those dielectric processes that are known to be influenced by crystallization itself. Namely, the α-crystalline process can be related to the existence of an interfacial region between amorphous and crystalline polymer, while the slow process can be related to the ionic conductivity in the amorphous matrix together with the existence of crystalline regions, exhibiting reduced conductivity, and providing the possibility of interfacial polarization.

Also, DSC is usually employed for the analysis of glass transition as well as crystallization processes. However, as already discussed in the Introduction, this technique is not ideally suited for the study of complex systems like polymers with disordered crystal structures. An example of the DSC thermogram for one of our samples is shown in Figure S3 of the Supplementary Materials, appearing as almost featureless, in agreement with most of the available literature results.

**Figure 7:**
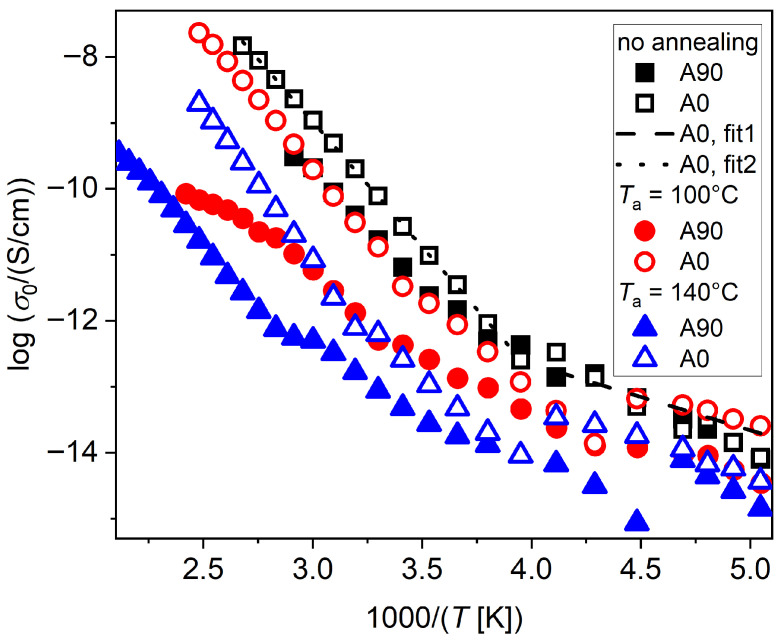
*dc* conductivity (*σ*_0_) as a function of temperature for chitosan samples annealed at different *T*_a_, by fitting to the tan*δ* function. A regime change in conductivity increase with temperature is observed. Arrhenius fittings are reported for the A0 sample with no annealing in the two regimes.

The presence of water and residual solvent molecules can be evidenced by the behavior of electric conductivity. Figure 7 shows the values of *dc* conductivity (*σ*_0_) obtained by the fitting of tan*δ*. At low temperatures, the conductivity increase with *T* is more moderate, with activation energy of around 20 kJ/mol, while over *T*_g_, the increase with temperature is steeper, with *E*_a_ around 70 kJ/mol. The conductivity exponent *n* turns from a fractional value of around 0.3–0.4 at the lowest temperatures, usually related to hopping conduction mechanisms, to around 0.9–1 at high temperature, indicating ohmic transport. Furthermore, a general trend of decreasing conductivity with increasing *T*_a_ is evident. Also, non-neutralized samples exhibit, in general, higher conductivity than neutralized ones, for the same *T*_a_. This conductivity transition was also observed for instance in Ref. [38] for the xanthan gum polysaccharide in hydrated conditions. According to such work, two conduction regimes of Arrhenius type can be present in hydrated samples. The first one, at lower temperature and with lower activation energy (around 15 kJ/mol), could be ascribed to ice water, while a second one is present at higher temperatures and low frequency, with a higher activation energy of around 80–90 kJ/mol. That one could be ascribed to *dc* conduction through adsorbed water molecules, for instance, hydrogen-bonded networks formed by the interfacial water, by peculiar conduction mechanisms involving proton transfer [39]. In particular, a proton may overcome the Coulomb potential energy barrier formed by the surrounding charges, and its transport could cross over from a sub-diffusive to a normal diffusion regime. These two regimes can be identified in conductivity spectra as a *dc* plateau at low frequency, where normal diffusion takes place, and an *ac* tail at high frequency, where the regime is sub-diffusive. This transition can be also evidenced on our spectral data, by considering the real part of the conductivity, *σ*′(*f*), at increasing temperatures. In Figure S4 of the Supplementary Materials, isothermal conductivity plots at increasing temperature values show a clear onset of the conductivity plateau at low frequency in correspondence to the temperature interval around *T*_g_.

**Figure 8:**
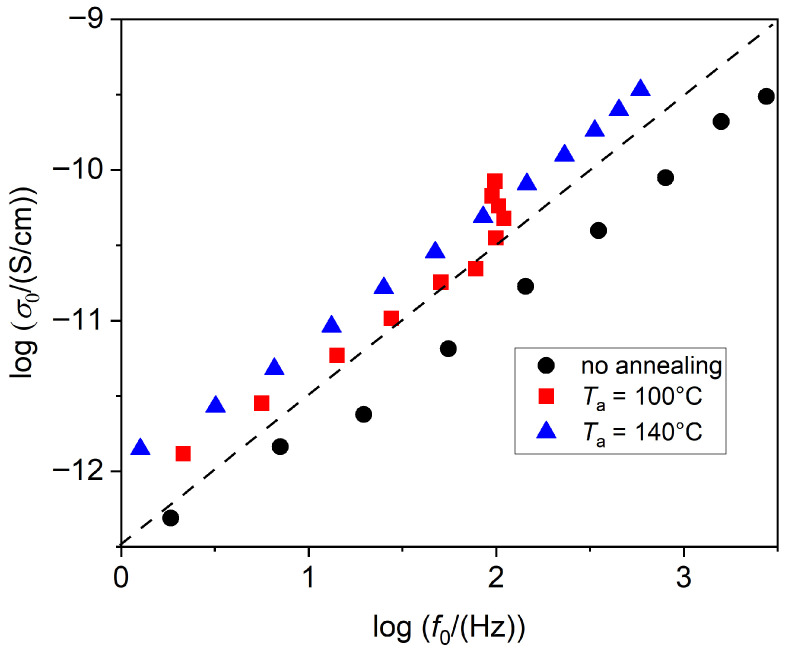
Correlation plot between the *dc* conductivity (*σ*_0_) and the relaxation frequency of the slow process of sample A90(1), for different *T*_a_. The dashed line indicates the slope of 1.

Let us now discuss the high-temperature (slow) process. Modeling of Maxwell–Wagner–Sillars (MWS) interfacial polarization, valid for inclusions with dielectric permittivity *ε*_1_ and conductivity *σ*_1_ into a matrix of dielectric permittivity *ε*_2_ and conductivity *σ*_2_, leads to a relaxation time [40]:

(8) $$\tau_{MWS}=\varepsilon_{0}\left(\frac{\varepsilon_{2}^{’}+A\phi ({\varepsilon_{1}^{’}-\varepsilon }_{2}^{’})}{\sigma_{2}+A\phi (\sigma_{1}{-\sigma }_{2})}\right)$$

where *A* is the depolarization factor of the inclusion (1/3 for spherical shape) and *ϕ* its volumetric fraction in the matrix. Therefore, increased conductivity leads to shorter relaxation times, as observed in our measurements. Indeed, a correlation between conductivity and relaxation frequency can be observed, as shown in Figure 8. In particular, relaxation frequency and conductivity are proportional, consistently with the case where the conductivity of the crystal phase is negligible compared to that of the amorphous phase, that is, when *σ*_1_ << *σ*_2_. The deviation for the case with *T*_a_ = 100 °C is probably due to the interference of the α_a_ process, which could not be discriminated from the slow process.

The slope changes and flattening of the Arrhenius plot for the slow process at high temperatures (Figure 5 and Figure 6, blue full symbols, as well as Figure S2 in the Supplementary Materials) seem more related to crystallization occurring during heating, necessary to record BDS isotherms at increasing temperatures, than to different polarization mechanisms. Indeed, the locations of slope changes seem to be correlated with the annealing temperatures. However, this aspect should be explored in more detail, also in combination with structural characterizations like, e.g., X-ray diffraction, and is out of the scope of the present paper.

Comparisons of our data with the literature results from Refs. [9,26] are presented in Figures S5 and S6 of the Supplementary Materials for neutralized and non-neutralized samples, respectively, using the largest open symbols. An interesting observation is that, along with β and σ (or slow) processes, an intermediate process is also detected in our measurements, which we identify as the α_c_ process, in a temperature range where the three processes can be obtained even from the fitting of single spectra. Additionally, in non-neutralized chitosan, where such a process was classified as a σ process in the literature, in our measurements the slow process appears at lower frequencies, leading to classification of the σ process from the literature as an α_c_ one in our terminology. This is also supported by the fact that the α_c_ process has a much weaker dependence on *T*_a_ than the slow process. Instead, the σ process from literature after annealing at 150 °C seems consistent with our slow process. The existence of a primary relaxation, either amorphous or crystalline, seems indeed plausible. This leads us to support our alternative assignment of relaxation processes.

## 5. Conclusions

In this work, we give evidence of the glass transition process in solution-cast chitosan samples, with two non-Arrhenian processes determined by BDS, where the temperature dependence can be described by a VFT trend [31]. Such processes are consistent with the relaxation of water slaved by the polymer matrix and the relaxation of the polymer plasticized by water, according to the interpretations in Ref. [24] for dextran and other polymers, at temperatures in the range from around −40 °C to +140 °C, depending on the water content and possibly on the crystalline structure. Since mutual influence is anticipated between water molecules and the polymer, this could facilitate chitosan crystallization even under ambient conditions, which instead would be inconsistent with the polymer *T*_g_ that becomes higher than room temperature when the water content decreases. Furthermore, we also observed a decrease in *dc* conductivity, related to the elimination of residual water at increasing temperatures [26], as well as to the increase in the crystalline fraction, which provides a higher hindrance to ionic transport. Such a decrease also influences the interfacial polarization at the amorphous/crystalline interface, slowing down the related relaxation process (MWS relaxation, here referred to as slow), which appears at the highest temperatures.

By using the same technique, we investigate the effect of thermal annealing (at temperature *T*_a_) through the possible role of the crystallization process along with the change in water content. The activation energy of the low-temperature relaxation process, related to local motions of the glycosidic rings, as well as that of the α_c_ process, related to confined amorphous chains, have a marked dependence on *T*_a_ that can be ascribed to the role of excess or hydration water on the polymer segmental mobility through the effect of hydrogen bonding. The influence of the neutralization process by alkaline treatment was also investigated, confirming that crystallization is hampered in non-neutralized samples, likely due to interchain Coulombic repulsion resulting from the abundance of protonated amines. To evidence the effects of the possible formation of different crystal phases, temperatures as high as 250 °C have been explored. However, crystallization in this regime could be influenced by chitosan decomposition, where the resulting effects on activation energy could be influenced by the tendency of the formed crystals to melt, degrade, or at least become more disordered. In conclusion, the detailed characterization of the glass transition for chitosan helps to better rationalize its crystallization behavior, in view of application of this biopolymer in food science and nanomedicine, and by its emerging piezoelectric properties [2,36], in the fields of sensors and actuators.

## Figures and Tables

**Table 1:** Activation energies *E*_a_ derived from Arrhenius fits, and glass transition temperatures *T*_g_ and dynamical fragility indexes *m* derived from VFT fits, of the different relaxation processes in solution-cast chitosan films with (A90) and without (A0) neutralization treatment, subjected or not to thermal annealing. Values of processes that could not be detected are indicated by “=”.

Sample	Process	*T*_a_ (°C)	log *f_∞_*	VFT *B* (K)	VFT *T*_0_ (K)	*T*_g_ (°C)	*m*	*E*_a_ (kJ/mol)
A90	βf	no ann.	=					=
A90	βf	100 °C	=					=
A90	βf	140 °C	=					=
A90	βf	200 °C	=					=
A90	βf	250 °C	=					=
A90	β	no ann.	16.40	5324	25.93	−126.8	23.3	
A90	β	100 °C	12.90	3168	70.75	−114.7	28.4	
A90	β	140 °C	12.0	3160	45.78	−134.7	22.1	
A90	β	200 °C	12.0	3083	54.19	−128.6	23.7	
A90	β	250 °C	10.87	2297	65.20	−135.0	25.9	
A90	αa	no ann.	10.03	2529	144.5	−43.0	34.5	
A90	αa *	100 °C	8.44	4185	86.0	−25.4	17.2	
A90	αa	140 °C	6.03	933.7	306.4	79.2	67.7	
A90	αa	200 °C	4.76	228.8	396.9	136.9	235.7	
A90	αa	250 °C	13.25	8645	84.0	44.7	21.8	
A90	αc	no ann.	=					=
A90	αc	100 °C	12.74					51.66
A90	αc	140 °C	12.7					58.14
A90	αc	200 °C	15.3					70.12
A90	αc	250 °C	16.4					77.31
A90	slow	no ann.	14.02					68.91
A90	slow *	100 °C	11.68					67.50
A90	slow	140 °C	12.83					88.94
A90	slow	200 °C	13.07					95.19
A90	slow	250 °C	17.52					142.9
A90	EP	no ann.	2.60					20.96
A90	EP	100 °C	−0.2					5.46
A90	EP	140 °C	0.599					12.77
A90	EP	200 °C	1.64					22.58
A90	EP	250 °C	6.77					73.09
A0	βf	no ann.	9.04					11.01
A0	βf	100 °C	=					=
A0	βf	140 °C	=					=
A0	β	no ann.	13.5					48.54
A0	β	100 °C	6.96	274.3	150.7	−110.3	130.3	
A0	β	140 °C	10.39	1922.3	70.5	−139.3	27.9	
A0	αa	no ann.	7.72	2340.8	144.7	−31.8	9.7	
A0	αa	100 °C	7.34	732.4	270.9	29.1	97.6	
A0	αa	140 °C	6.20	467.1	305.5	54.9	130.9	
A0	αc	no ann.	18.66					85.48
A0	αc	100 °C	18.13					82.47
A0	αc	140 °C	12.64					120.7
A0	slow	no ann.	5.70					29.93
A0	slow	100 °C	9.99					59.79
A0	slow	140 °C	19.38					54.24
A0	EP	no ann.	7.73					50.29
A0	EP	100 °C	6.80					44.80
A0	EP	140 °C	7.59					62.03

* The α_a_ and slow processes could not be discriminated by fitting.

## Data Availability

The original contributions presented in this study are included in the article/Supplementary Material. Further inquiries can be directed to the corresponding author.

## Supplementary Materials

The following supporting information can be downloaded at https://www.mdpi.com/article/10.3390/polym17202758/s1, Figure S1: Thermogravimetric analysis (TGA) thermograms for a neutralized chitosan film compared to the non-neutralized one; Figure S2: Relaxation plots for the neutralized samples, subjected to multiple annealings up to 250 °C; Figure S3: DSC scans of a neutralized chitosan sample; Figure S4: Real part of conductivity (*σ*′) isotherms at increasing temperatures; Figure S5: Relaxation plots for the neutralized samples, subjected to multiple annealings up to 140 °C, with the additions of the literature data; Figure S6: Relaxation plots for the non-neutralized samples, subjected to multiple annealings up to 140 °C, with the additions of the literature data.

## Abbreviations

The following abbreviations are used in this manuscript:

DSC	Differential Scanning Calorimetry
DMTA	Dynamic Mechanical Thermal Analysis
BDS	Broadband Dielectric Spectroscopy
PVDF	Poly(Vinylidene Fluoride)
VFT	Vogel–Fulcher–Tammann
MWS	Maxwell–Wagner–Sillars
XRD	X-Ray Diffraction
EP	Electrode Polarization

## References

[1] Rinaudo M. (2006). Chitin and chitosan: Properties and applications. Prog. Polym. Sci..

[2] De Marzo G., Mastronardi V.M., Algieri L., Vergari F., Pisano F., Fachechi L., Marras S., Natta L., Spagnolo B., Brunetti V. (2022). Sustainable, flexible, and biocompatible enhanced piezoelectric chitosan thin film for compliant piezosensors for human health. Adv. Electron. Mater..

[3] Baklagina Y.G., Klechkovskaya V.V., Kononova S.V., Petrova V.A., Poshina D.N., Orekhov A.S., Skorik Y.A. (2018). Polymorphic modifications of chitosan. Crystallogr. Rep..

[4] Ogawa K., Yui T., Okuyama K. (2004). Three D structures of chitosan. Int. J. Biol. Macromol..

[5] Kim K.M., Son J.H., Kim S.-K., Weller C.L., Hanna M.A. (2006). Properties of chitosan films as a function of pH and solvent type. J. Food Sci..

[6] Papa S., Montorsi M., Ferrari L.M., Arrighetti L., Rodriguez-Tinoco C., Capaccioli S., Greco F., Labardi M. (2025). Optimizing piezoelectric performance in chitosan: The impact of fabrication process and crystalline content. Small Sci..

[7] Qiao C., Ma X., Zhang J., Yao J. (2019). Effect of hydration on water state, glass transition dynamics and crystalline structure in chitosan films. Carbohydr. Polym..

[8] Dong Y., Ruan Y., Wang H., Zhao Y., Bi D. (2004). Studies on glass transition temperature of chitosan with four techniques. J. Appl. Polym. Sci..

[9] Gonzalez-Campos J.B., Prokhorov E., Luna-Barcenas G., Fonseca-Garcia A., Sanchez I.C. (2009). Dielectric relaxations of chitosan: The effect of water on the α-relaxation and the glass transition temperature. J. Polym. Sci. Part B Polym. Phys..

[10] Quijada-Garrido I., Iglesias-Gonzalez V., Mazon-Arechederra J.M., Barrales-Rienda J.M. (2007). The role played by the interactions of small molecules with chitosan and their transition temperatures. Glass-forming liquids: 1,2,3-Propantriol (glycerol). Carbohydr. Polym..

[11] Ratto J., Hatakeyama T., Blumstein R.B. (1995). Differential scanning calorimetry investigation of phase transitions in water/chitosan systems. Polymer.

[12] Lazaridou A., Biliaderis C.G. (2002). Thermophysical properties of chitosan, chitosan-starch and chitosan-pullulan films near the glass transition. Carbohydr. Polym..

[13] Madeleine-Perdrillat C., Karbowiak T., Debeaufort F., Delmotte L., Vaulot C., Champion D. (2016). Effect of hydration on molecular dynamics and structure in chitosan films. Food Hydrocoll..

[14] Bonilla J., Bittante A.M.Q.B., Sobral P.J.A. (2017). Thermal analysis of gelatin–chitosan edible film mixed with plant ethanolic extracts. J. Therm. Anal. Calorim..

[15] Hosseini S.F., Rezaei M., Zandi M., Ghavi F. (2013). Preparation and functional properties of fish gelatin–chitosan blend edible films. Food Chem..

[16] Qiao C., Ma X., Wang X., Liu L. (2021). Structure and properties of chitosan films: Effect of the type of solvent acid. LWT—Food Sci. Technol..

[17] Ogura K., Kanamoto T., Itoh M., Miyashiro H., Tanaka K. (1980). Dynamic mechanical behavior of chitin and chitosan. Polym. Bull..

[18] Sakurai K., Maegawa T., Takahashi T. (2000). Glass transition temperature of chitosan and miscibility of chitosan/poly(N-vinyl pyrrolidone) blends. Polymer.

[19] Kumar-Krishnan S., Prokhorov E., Ramírez M., Hernandez-Landaverde M.A., Zarate-Triviño D.G., Kovalenko Y., Sanchez I.C., Luna-Bárcenas G. (2014). Novel gigahertz frequency dielectric relaxations in chitosan films. Soft Matter.

[20] Bouharras F.E., Labardi M., Tombari E., Capaccioli S., Raihane M., Améduri B. (2023). Dielectric characterization of core-shell structured poly(vinylidene fluoride)-grafted-BaTiO_3_ nanocomposites. Polymers.

[21] Rekik H., Ghallabi Z., Royaud I., Arous M., Seytre G., Boiteux G., Kallel A. (2013). Dielectric relaxation behaviour in semi-crystalline polyvinylidene fluoride (PVDF)/TiO_2_ nanocomposites. Compos. Part B.

[22] Li Y.Q., Zhang C.X., Jia P., Zhang Y., Lin L., Yan Z.B., Zhou X.H., Liu J.-M. (2018). Dielectric relaxation of interfacial polarizable molecules in chitosan ice-hydrogel materials. J. Mater..

[23] Khodadadi S., Roh J.H., Kisliuk A., Mamontov E., Tyagi M., Woodson S.A., Briber R.M., Sokolov A.P. (2010). Dynamics of biological macromolecules: Not a simple slaving by hydration water. Biophys. J..

[24] Combarro Palacios I., Olsson C., Kamma-Lorger C.S., Swenson J., Cerveny S. (2019). Motions of water and solutes—Slaving versus plasticization phenomena. J. Chem. Phys..

[25] Nogalez A., Ezquerra T.A., Rueda D.R., Martinez F., Retuert J. (1997). Influence of water on the dielectric behaviour of chitosan films. Colloid Polym. Sci..

[26] Viciosa M.T., Dionisio M., Silva R.M., Reis R.L., Mano J.F. (2004). Molecular motions in chitosan studied by dielectric relaxation spectroscopy. Biomacromolecules.

[27] Meissner D., Einfeldt J., Kwasniewski A. (2000). Contributions to the molecular origin of the dielectric relaxation processes in polysaccharides—The low temperature range. J. Non-Cryst. Solids.

[28] Einfeldt J., Meissner D., Kwasniewski A. (2003). Contributions to the molecular origin of the dielectric relaxation processes in polysaccharides—The high temperature range. J. Non-Cryst. Solids.

[29] Viciosa M.T., Dionisio M., Mano J.F. (2006). Dielectric characterization of neutralized and non-neutralized chitosan upon drying. Biopolymers.

[30] Jonscher A.K. (1996). Universal Relaxation Law.

[31] Kremer F., Schoenhals A. (2003). Broadband Dielectric Spectroscopy.

[32] Lewis T.J. (1994). Nanometric dielectrics. IEEE Trans. Dielectr. Electr. Insul..

[33] Kaminski K., Kaminska E., Ngai K.L., Paluch M., Wlodarczyk P., Kasprzycka A., Szeja W. (2009). Identifying the origins of two secondary relaxations in polysaccharides. J. Phys. Chem. B.

[34] Naito P.K., Ogawa Y., Kimura S., Iwata T., Wada M. (2015). Crystal transition from hydrated chitosan and chitosan/monocarboxylic acid complex to anhydrous chitosan investigated by X-ray diffraction. J. Polym. Sci. B Polym. Phys..

[35] Takara E.A., Marchese J., Ochoa N.A. (2015). NaOH treatment of chitosan films: Impact on macromolecular structure and film properties. Carbohydr. Polym..

[36] Nicoletti J., Puppulin L., Routurier J., Frroku S., Loudhaief N., Crestini C., Perosa A., Selva M., Gigli M., Back M. (2025). Enhanced piezoelectricity in sustainable-by-design chitosan nanocomposite soft thin films for green sensors. ACS Nano.

[37] Podgorbunskikh E., Kuskov T., Rychkov D., Lomovskii O., Bychkov A. (2022). Mechanical amorphization of chitosan with different molecular weights. Polymers.

[38] Tegopoulos S.N., Papagiannopoulos A., Kyritsis A. (2024). Hydration effects on thermal transitions and molecular mobility in Xanthan gum polysaccharides. Phys. Chem. Chem. Phys..

[39] Popov I., Zhu Z., Singh H., Abdullah M., Sacci R.L., Mamontov E., Damron J.T., Gainaru C., Paddison S.J., Sokolov A.P. (2024). Mechanisms of proton transport in aqueous acid solutions. Cell Rep. Phys. Sci..

[40] Pelster R., Simon U. (1999). Nanodispersions of conducting particles: Preparation, microstructure and dielectric properties. Colloid Polym. Sci..

