# Supplementary material for: Glass Transition and Crystallization of Chitosan Investigated by Broadband Dielectric Spectroscopy
Source: Polymers (Basel). 2025 Oct 15;17(20):2758. doi: 10.3390/polym17202758 (PMC12567186; doi:10.3390/polym17202758)
Supplement: Supplementary file 1 [file polymers-17-02758-s001.zip › polymers-3808159-supplementary.pdf]

Glass transition and crystallization of chitosan investigated by broadband dielectric spectroscopy

M. Labardi,[1,*] M. Montorsi,[1,2] S. Papa,[3] L.M. Ferrari,[3] F. Greco,[3,4] G. Scarioni,[5] and S. Capaccioli[1,5]

[1] *CNR-IPCF, Sede Secondaria di Pisa, Largo Pontecorvo 3, 56127 Pisa, Italy*

[2] *College of Physics and Optoelectronic Engineering, Shenzhen University, 518060 Shenzhen, People's Republic of China*

[3] *The Biorobotics Institute and Dept. of Excellence in Robotics & AI, Scuola Superiore Sant'Anna, Viale R. Piaggio 34, 56025 Pontedera, Italy*

[4] *Interdisciplinary Center on Sustainability and Climate, Scuola Superiore Sant'Anna, Piazza Martiri della Libertà 33, 56127 Pisa, Italy*

[5] *Physics Department, University of Pisa, Largo Pontecorvo 3, 56127 Pisa, Italy*

* Corresponding author, email massimiliano.labardi@cnr.it

Supplementary Materials.

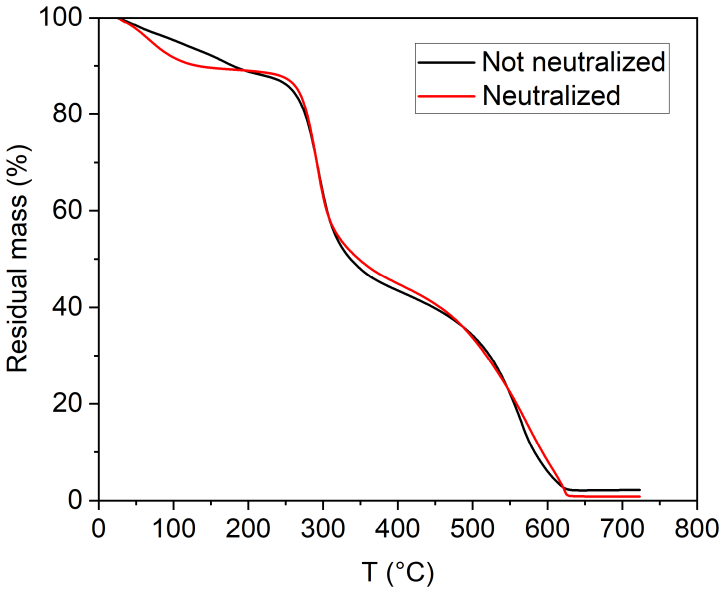

Figure S1. Thermogravimetric analysis (TGA) thermograms for a neutralized chitosan film compared to the non-neutralized one.

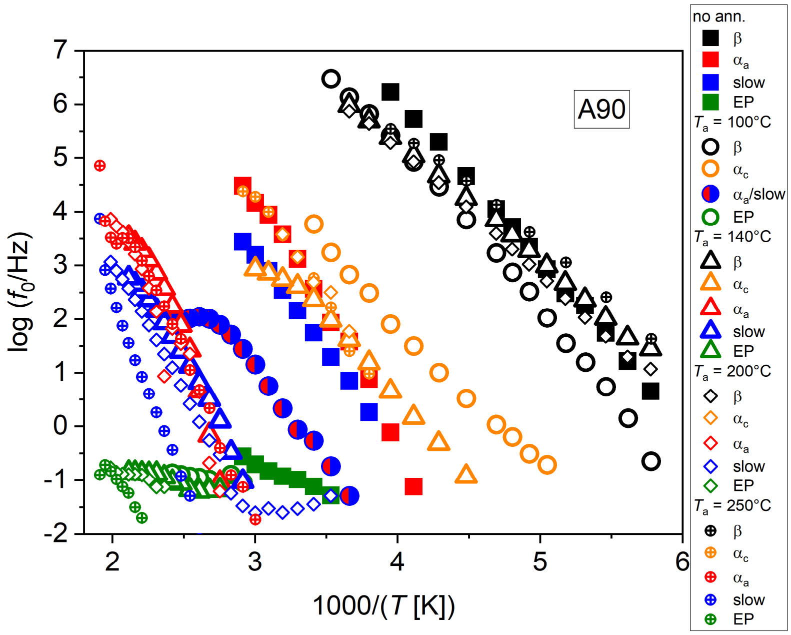

Figure S2. Relaxation plots for the neutralized samples, subjected to multiple annealings up to 250°C. All values were obtained by fitting of $\tan\delta$ data.

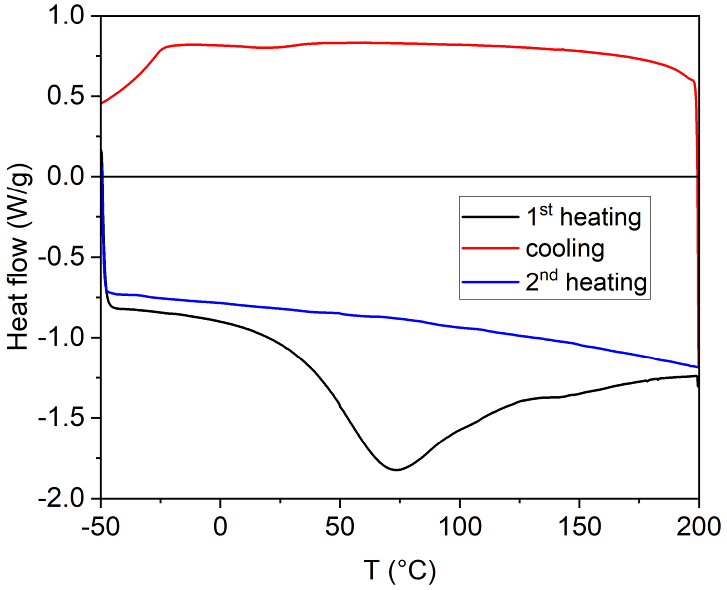

Figure S3. DSC scans of a neutralized chitosan sample. The first peak is related to water evaporation. No clear features related to glass transition or cold crystallization can be evinced, most likely due to a high degree of disorder of the crystalline formations.

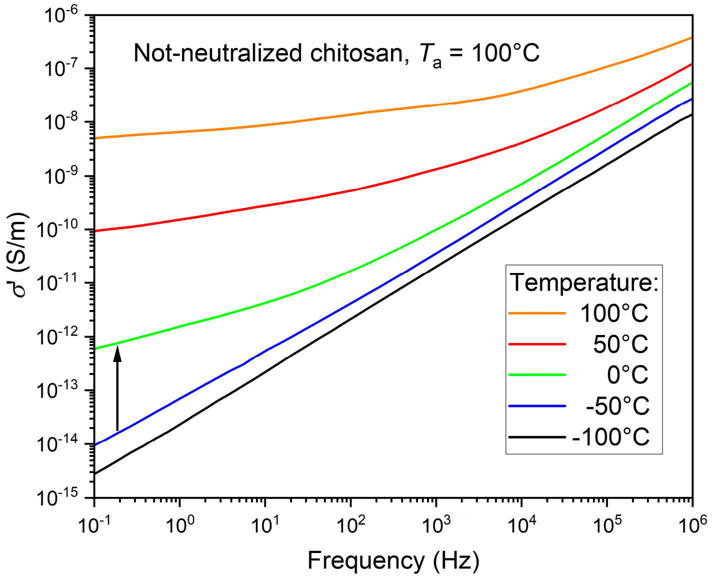

Figure S4. Real part of conductivity (σ') isotherms at increasing temperatures. The arrow highlights the regime change in conductivity increase with temperature.

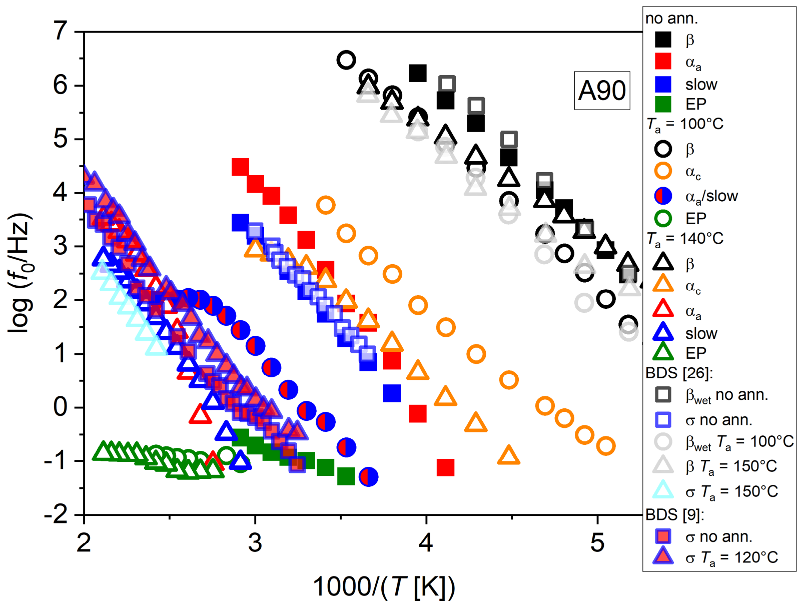

Figure S5. Relaxation plots for the neutralized samples, subjected to multiple annealings up to 140°C, with added data from the literature (bigger open symbols, from Refs. [9] and [26]). The literature data adapted with permission from the following: [9] Gonzales-Campos et al., *Journal of Polymer Science: Part B: Polymer Physics* 2009, 47, 2259, Copyright 2009 Wiley Periodicals, Inc.; [26] Viciosa et al., *Biomacromolecules* 2004, 5, 2073; Copyright 2004 American Chemical Society.

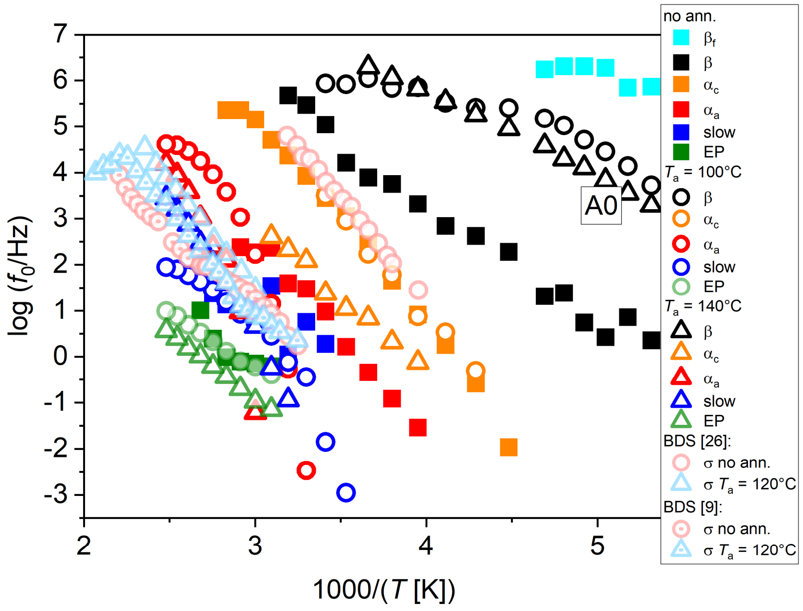

Figure S6. Relaxation plots for the non-neutralized samples, subjected to multiple annealings up to 140°C, with added data from the literature (bigger open symbols, from Refs. [9] and [26]). The literature data adapted with permission from the following: [9] Gonzales-Campos et al., *Journal of Polymer Science: Part B: Polymer Physics* 2009, 47, 2259, Copyright 2009 Wiley Periodicals, Inc.; [26] Viciosa et al., *Biomacromolecules* 2004, 5, 2073; Copyright 2004 American Chemical Society.
